# The Application of Nano Silver Argitos as a Final Root Canal Irrigation for the Treatment of Pulpitis and Apical Periodontitis. In Vitro Study

**DOI:** 10.3390/nano12020248

**Published:** 2022-01-13

**Authors:** Svetlana Razumova, Anzhela Brago, Dimitriy Serebrov, Haydar Barakat, Yuliya Kozlova, Ammar Howijieh, Zoya Guryeva, Yulianna Enina, Vasiliy Troitskiy

**Affiliations:** 1 Department of Propedeutics of Dental Diseases, Medical Institute, Peoples’ Friendship University of Russia (RUDN University), 6 Miklukho-Maklaya Street, 117198 Moscow, Russian Federation; razumova-sn@rudn.ru (S.R.); brago-as@rudn.ru (A.B.); serebrov-dv@rudn.ru (D.S.); kozlova-yus@rudn.ru (Y.K.); 1072168012@rudn.ru (A.H.); guryeva-za@rudn.ru (Z.G.); 2 Department of Propedeutics of Dental Diseases, I.M. Sechenov First Moscow State Medical University (Sechenov University), Bolshaya Pirogovskaya Street 2, Building 4, 119435 Moscow, Russian Federation; yuliannatr@mail.ru; 3 Department of Infectious Diseases, Institute of Clinical Medicine, I.M. Sechenov First Moscow State Medical University (Sechenov University), Bolshaya Pirogovskaya Street 2, Building 4, 119435 Moscow, Russian Federation; Troickii_vasilii@mail.ru

**Keywords:** root canal treatment, nano-silver, dentinal surface, irrigants

## Abstract

Background: Endodontic treatment of various forms of pulpitis with variations of root canal system anatomy should be performed with high quality. The use of various antibacterial agents is aimed at maintaining the success of endodontic treatment. The aim of this study was to evaluate the penetration and fixation of the nano-silver solution on the dentinal surface during endodontic treatment. Materials and methods: the study was carried out on 70 extracted single-rooted teeth, randomly divided into two groups. In the teeth of the first group, the smear layer was removed after canal preparation with 17% EDTA solution; in the second group, the smear layer was not removed. In both groups, for the final treatment of the canal, a colloidal 1% solution of нанo серебра nanosilver was used. Samples were cut and prepared for analysis using micro-CT, scanning electron microscopy (SEM), X-ray microanalysis and energy dispersive spectrometry (elemental mapping). Results: in 100% of cases in groups of teeth with a preserved smear layer, the ability of a 1% colloidal solution of nanosilver with particles of 1–2 nm to be fixed on dentin with a removed and preserved smear layer and to leave a film on the dentinal surface was established. In the samples with removed smear layer, silver was found in 73.5% of cases. Conclusion: The nano-silver solution with a particle size of 1–2 nm proved its ability to penetrate the dentinal surfaces and create a final film covering the dentinal surface of the root canal before applying the sealer.

## 1. Introduction

The long-term preservation of the effect, after performing a high-quality endodontic treatment, is facilitated by effective mechanical and medical treatment of the root canal and the tightness of the post-endodontic restoration [1]. For medical treatment of the root canal, a 1–3% solution of sodium hypochlorite is traditionally used. According to various authors, the use of this solution ensures the success of endodontic treatment in 40–75% of cases [2,3]. However, in the treatment of necrotic forms of pulpitis and in cases of repeated endodontic treatment, it becomes necessary to use calcium hydroxide, chlorhexidine, MTAD (mixture of Doxycycline, citric acid, and a detergent) and other antiseptics [1,2,3].

The problems that face clinicians during endodontic treatment can be explained by treating teeth with complex root canal anatomy [4,5], or teeth that have cross-sectional root canal shape differs from the round or oval ones [6] or treatment with the apical third of the root canal [7]. The goal of endodontic treatment is to preserve the long-term results of antibacterial treatment of the root canal walls. Sodium hypochlorite (NaОCl) copes with the task of antibacterial root canal treatment. The use of other antiseptics-chlorhexidine, MTAD and others-is aimed at maintaining a long-term antibacterial effect or affecting microflora that is not sensitive to sodium hypochlorite. Using of an antibacterial agent is generally short-lived [8]. An antibacterial drug that will maintain this property for a long time in the root canal will ensure 100% success of endodontic treatment.

Silver is a safe inorganic, non-toxic, antibacterial agent used for centuries and can kill about 650 types of microorganisms [9]. Silver ions and silver-based compounds including silver nanoparticles are highly toxic to microorganisms but have low toxicity towards host cells [10]. Silver solutions have shown excellent antibacterial effects. However, their use has been limited due to their high ability to stain the teeth in a dark color.

Currently, there are several types of solutions on the market that contain silver nanoparticles and can be used in endodontics. The biocompatibility of a root canal irrigant based on positively charged imidazolium with an ionic liquid protected by a nanosilver solution (AgNPs) was studied by Nabavizadeh et al. The researchers concluded that AgNPs is a tissue-compatible agent when compared to NaОCl and chlorhexidine [11]. Volkov & Mitronin studied the antibacterial activity of colloidal silver during endonanophoresis and they proved the clinical efficacy of Poviargol hydrocolloidal silver solution in endodontics [12].

González-Luna et al. evaluated the root canal treatment with a nano-silver solution 10 nm particle size. The results obtained showed that nanoparticles with a size of 10 nm and 2.25% sodium hypochlorite were effective for the elimination of Enterococcus faecalis, and there was no significant difference between the irrigants. In addition, silver nanoparticles had a good ability to remove the smear layer. The authors concluded that silver nanoparticles may be a good option for removing Enterococcus faecalis from root canals [13].

Rodrigues et al. (2018) evaluated the antimicrobial effect of an irrigant containing silver nanoparticles in an aqueous carrier in comparison with sodium hypochlorite and chlorhexidine solutions against E. faecalis biofilm and infected dentinal tubules. The study was conducted on bovine teeth. The researchers concluded that the AgNP irrigant was not as effective against E. faecalis compared to solutions commonly used in root canal treatment. NaOCl is suitable as an irrigant because it was effective in destroying biofilm and killing bacteria in biofilms and dentinal tubules [14].

Generali et al. (2020) investigated the cytotoxicity and antimicrobial activity of two solutions of irrigants based on silver citrate. The cytotoxicity of various concentrations (0.25%, 0.5%, 1%, 2.5%, 5%) of both solutions (BioAKT and BioAKT Endo) was assessed on mouse fibroblasts L-929 using MTT analysis. Both silver citrate solutions showed > 70% viability of murine fibroblasts when diluted to 0.25% and 0.5%. At higher concentrations, they were extremely cytotoxic. FT-IR spectroscopy measurements of both liquids showed the same spectra, indicating similar chemical characteristics. Both solutions used as root canal irrigants exhibited significant antimicrobial activity and low cytocompatibility at dilutions of more than 0.5% [15].

Recently, а new colloidal solution of silver nanoparticles, colorless and odorless (ARGITOS) was developed using advanced nanobiotechnologies and “green chemistry” methods and consists of silver, sodium peroxide (stabilizer), bidistillate. The solution was prepared in concentrations (1000–10,000 ppm) of particles 1–2 nm in size. The drug has a high bactericidal activity against gram-positive and gram-negative bacteria, including tubercle bacillus, virucidal activity against poliomyelitis, hepatitis A, HIV infection. Effectively it inhibits rod microorganisms (salmonellosis, Listeria, Pseudomonas aeruginosa) [16].

According to the results of many studies, the use of various products based on a hydrocolloidal solution of nano-silver gives a positive antibacterial effect. The effectiveness of using nano-silver products as a final irrigant, for the formation of a silver film at the sealer-dentin interface, has been insufficiently studied. Therefore, this study aimed to evaluate the penetration and fixation of the nano-silver solution on the dentinal surface during endodontic treatment in the presence or removal of the smear layer.

## 2. Materials and Methods

This in vitro study included 70 single-rooted teeth extracted for periodontal indications. The teeth were disinfected in a 7% sodium hypochlorite solution for an hour, then washed with water and prepared for endodontic treatment. Next, a standard mechanical treatment of the root canal was carried out using hand instruments to size 15 (K-file, H-file) (Mani, INC, Tochigi, Japan) and then with profile machine instruments (Dentsply Sirona, USA), MTWo (VDW, Germany), and Protaper (Dentsply® Sirona, West Philadelphia USA) canal prepared to the size of apical master file No. 35–40. For irrigation, a 3% sodium hypochlorite solution (Omega dent, Moscow, Russia) was used with passive activation with ultrasound activation after each file (Woodpecker, Guilin, China) with endodontic tips E1. Then all the teeth were divided randomly into two groups, 35 teeth each. Group 1 teeth were washed with water and dried, then a 17% EDTA solution (EDTA 17% (META Biomed, Cheongju-si, Korea) was injected into the root canal for 1 min, the root canal was washed with water for 1 min and dried with paper pins. In the teeth of the second group, the smeared layer was not removed by EDTA. The canals were washed with water and at the end, the root canals of the teeth of both groups were treated with a 1% solution of nano-silver (Argitos ARGITOS 1% solution of colloidal nano-silver with particles of 1–2 nm NANOSPHERE company, Moscow, Russia). In each group, one sample was left for control, not treated with 1% solution.

In each group, for analyzing the thickness and penetration of silver nanoparticles into the root canal four samples were selected randomly to be examined on a v |tome| x m300 phoenix tomographic by X-ray computed tomography with the parameters presented in Table 1 and Table 2.

After micro-CT scanning, the samples were cut longitudinally. The remaining 30 samples of each group were filled with the injection method using thermoplasticized gutta-percha, and then the root canal was cut into three parts in the horizontal plane. All samples (longitudinal sections and horizontal sections) of both groups were scanned for the presence of silver by energy dispersive spectroscopy analysis (elemental mapping) under low vacuum conditions. Scanning parameters: Accelerating voltage 30 kV, beam current 0.63 nA on a Thermo Scientific Quattro S setup with energy dispersive spectrometry Bruker XFlash 6160.

Then, on a Thermo Scientific Helios G4 PFIB UXe setup with an EDAX Octane Elite energy dispersive spectrometry, the procedure was carried out to study the sample structure using a cross-section, energy dispersive spectrometry analysis (elemental mapping). Accelerating voltage of the electron column: 30 kV, beam current 1.6 nA, Accelerating voltage of the ion column: 12 kV (deposition of a protective platinum layer) at a beam of 0.33 nA, 30 kV (etching of the cross-section) 15 nA, (polishing of the cross-section) 1 nA. Before the procedure, a thin layer of gold was applied to the surface of the sample using a magnetron to ensure the drainage of the surface charge. Then all samples were visually assessed using macro photography to analyze the silver oxidation zone on the inner wall of the root canal.

Statistical analysis was conducted using SPSS v 22 (SPSS, Inc., Chicago, IL, USA). For the comparison between groups, the Mann–Whitney U test was used with a p-value set at 0.05.

## 3. Results

**Figure 1:**
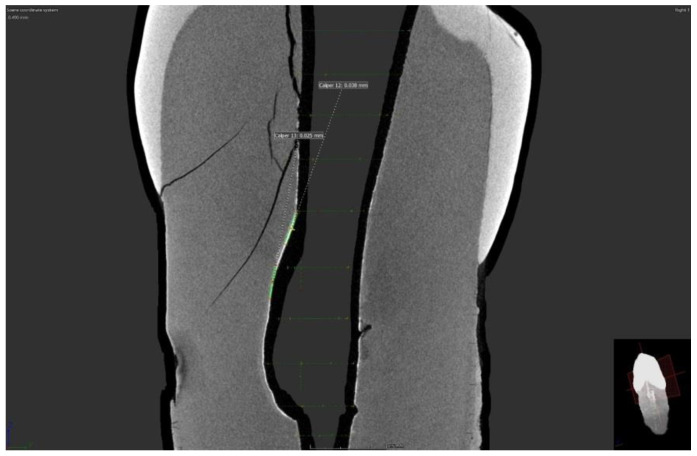
Micro CT scan of sample with removed smear layer.

**Figure 2:**
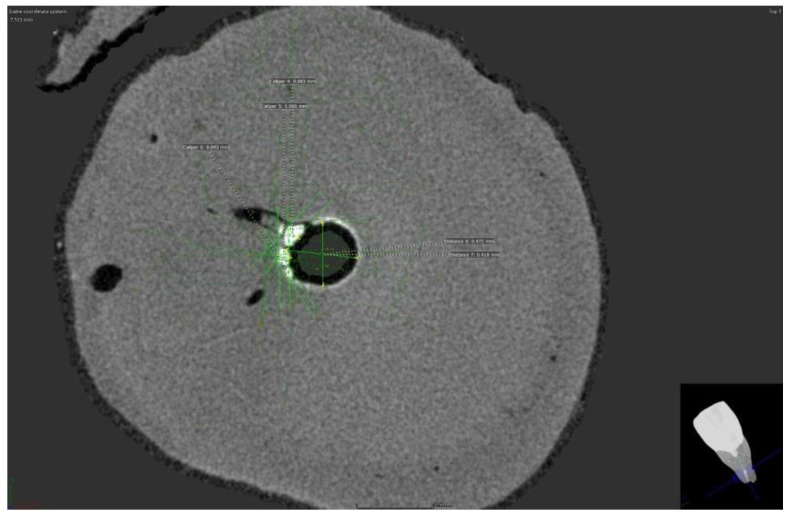
Micro CT scan of sample with preserved smear layer.

Micro-CT scans showed the presence of a silver film on all samples of both groups with removed and preserved smear layers. However, imaging of these films shows an uneven distribution of silver film over the dentinal surface (Figure 1 and Figure 2).

A series of horizontal vertical images showed the uneven distribution of the silver film over the inner surface of the root canal, but in some scans, it was even possible to measure the penetration depth or the thickness of the silver film. In samples with a removed smear layer, the penetration depth ranged from 0.023 to 0.028 nm. Moreover, in the group with preserved smear layer, the thickness of the silver film ranged from 0.023 to 0.066 nm in different scans.

A study on longitudinal sections of samples in groups with a preserved smear layer (n = 4) and removed smear layer (n = 4), by the method of energy dispersive spectral analysis (elemental mapping), showed that on the inner walls of root canals treated with colloidal solution, silver was found in 100% (n = 8) cases. On the inner wall of the root canal of the control samples of both groups, silver was not found (n = 2).

**Figure 3:**
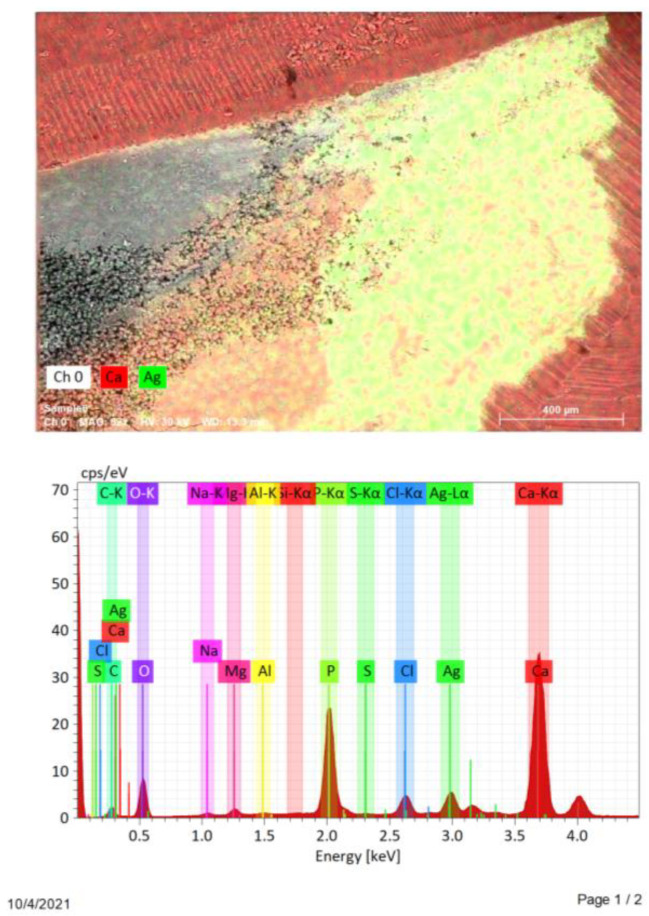
Scans of longitudinal sections of specimens group with removed smear layer.

**Figure 4:**
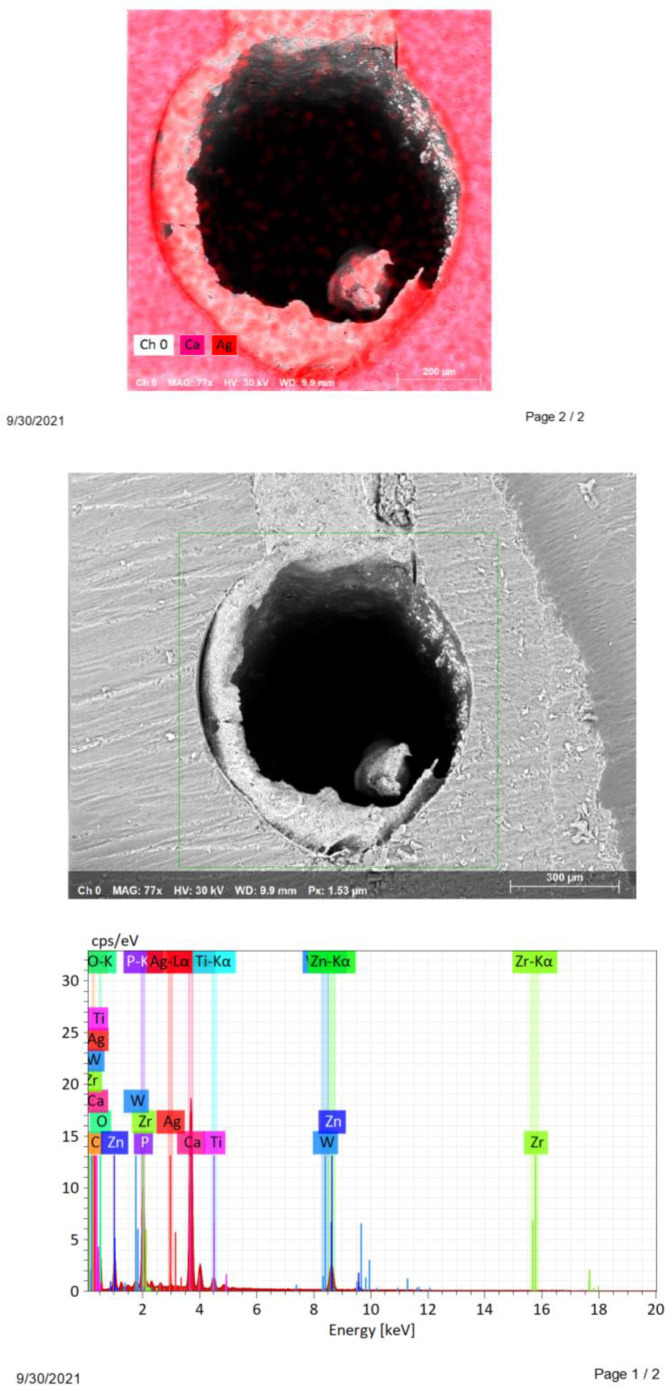
Scans of cross-sections of specimens group with preserved smear layer.

In the cross-sectional samples in the group with removed smear layer, the presence of silver was found in 33.3% (n = 10) cases, and in the samples with preserved smear layer, silver was detected in 53.3% (n = 16) cases. The data are presented in Table 3 and in Figure 3 and Figure 4.

**Figure 5:**
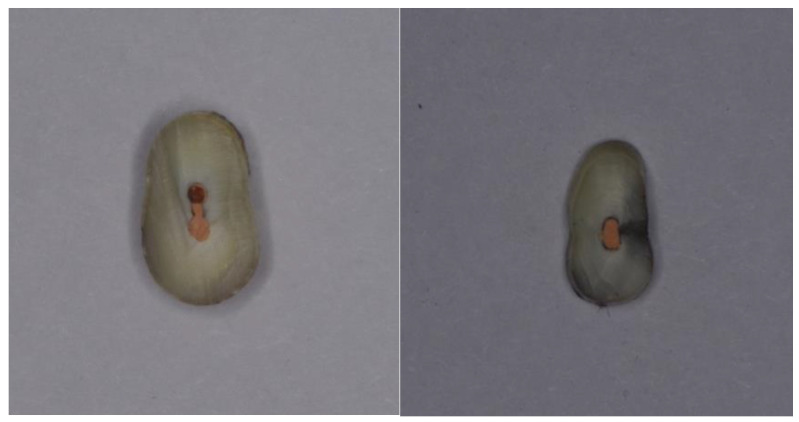
Samples with preserved smear layer.

**Figure 6:**
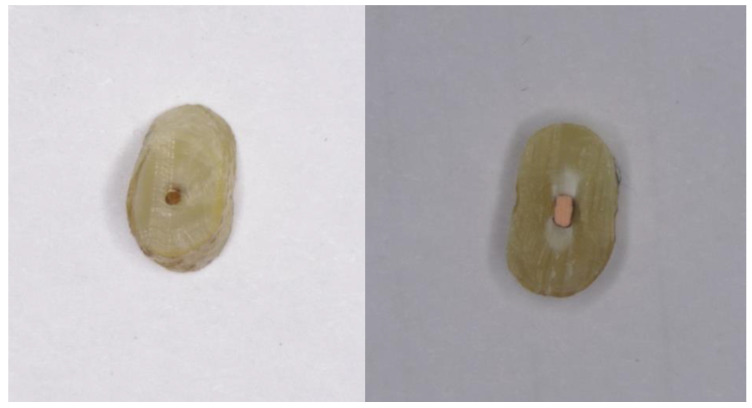
Samples with removed smear layer.

Storing the samples without airtight packaging has caused oxidation and visualized the silver used to treat the canals. In samples with preserved smear layer, silver was visualized on all tooth samples 100% (n = 30). In teeth with removed smear layer, silver was visualized on 25 samples (73.5%). The data was presented in Figure 5 and Figure 6.

## 4. Discussion

Successful endodontic treatment depends on the quality of mechanical and medical root canal treatment. This study was the first to evaluate the effectiveness of nano-silver solution as a final irrigant in penetrating the dentinal surfaces and creating a silver film in the inner wall of root canals. The preservation of the nano-silver film on the dentinal surfaces forms a barrier to the penetration of microorganisms into the dentinal tubules and peri-apically. Small particles of nano-silver 1–2 nm could have a long-term antibacterial effect without staining the tooth tissue.

In this study we evaluated the nanosilver Argitos solution (size 1–2 nm), Argitos solution showed a high ability to impregnate the smear layer. This is confirmed by the study by González-Luna [13] in which they found that 10 nm silver nanoparticles have a pronounced antimicrobial effect against *E. faecalis* and suggested using silver compounds for root canal finishing.

Ioannidis et al. (2019) studied the antimicrobial efficacy of silver nanoparticles (AgNPs) synthesized on an aqueous graphene oxide (GO) (Ag-GO) matrix. As a result of the experiment, it was found that the efficiency of killing microorganisms with 2.5% NaOCl solution was higher in comparison with the experimental groups. The maximum destruction of the biofilm on the surface of the dentinal tubules was achieved with a 2.5% NaOCl solution; however, Ag-GO caused a significant decrease in the total amount of biofilm volumes compared to other experimental groups. The study confirmed the long-term antibacterial effect of nanosilver particles, which helps to reduce the total amount of biofilm volumes on the surface of root canal walls. [17].

Tonini et al. (2020) studied the antibacterial activity of the BioACT solution which is based on silver citrate in comparison with EDTA and sodium hypochlorite, and their efficiency of removing the smear layer and the penetration of the sealer. BioACT and EDTA were the most effective irrigants at removing the smear layer and facilitating the penetration of the sealer into the dentin. In the apical part of the root canal, BioACT showed significantly better results when removing the smear layer and when sealing. BioACT and sodium hypochlorite showed comparable antibacterial effects. The researchers concluded that BioACT provides reliable penetration of the sealer into the apical part of the root canal system and has significant antibacterial properties [18].

The properties of the nano-silver solution in this study to penetrate the smear layer and leave a film on the dentinal surface will create a barrier to the penetration of microorganisms into the canal.

## 5. Conclusions

Within the limits of this study, the presence of a smear layer promotes a better fixation of nanosilver particles on the dentinal surface, which intern could create a final film covering the dentinal surface of the root canal before applying the sealer. This will allow for long-term preservation of endodontic treatment results.

## Figures and Tables

**Table 1:** GE v |tome| x m300 parameters.

N	Characteristic	Value
1	Voltage, kV	300/180
2	Power, W	500/20
3	Distinction of details, microns	<1.0/<0.5
4	Detector resolution, MPix	4
5	Max. sample dimensions, mm	500 × 600
6	Volume of 3D-tomography, mm	290 × 400
7	Max. manipulator load, kg	Up to 20

**Table 2:** Parameters scanning of samples.

N	Parameters Scanning	Value
1	Voltage, kV	130
2	Current, μA	70
3	Power, W	9.1
4	Focal spot size, μm	9.1
5	Shooting time, min	30
6	Voxel size, μm	10
7	Number of projections, pcs.	2400

**Table 3:** Results of silver detection in groups of samples with removed and preserved smear layer.

Sample Groups/Quantity		Energy Dispersive Spectral Analysis (Elemental Mapping)		*p*-Value	MACRO Photography		*p*-Value
		Group of Samples with Removed Smear Layer	Group of Samples with Preserved Smear Layer		Group of Samples with Removed Smear Layer	Group of Samples with Preserved Smear Layer	
Longitudinal sections	Ag−	(0) not found	(0) not found	1.00	(0) not found	(0) not found	1.00
	Ag+	(4) silver found (100%)	(4) silver found (100%)		(4) silver found (100%)	(4) silver found (100%)	
Cross sections	Ag−	(20) not found (66.7%)	(14) not found (46.7%)	0.12	(9) not found (30%)	(0) not found (0%)	0.01
	Ag+	(10) silver found (33.3%)	(16) silver found (53.3%)		(21) silver found (70%)	(30) silver found (100%)	
Control longitudinal (1 sample)		silver not found (1)	silver not found (1)	1.00	silver not found (1)	silver not found (1)	1.00
Total silver found		14 (41.2%)	20 (58.8%)	0.14	25 (73.5%)	34 (100%)	0.001
